# A case report of partial bilateral hind limb adactyly in a male lamb

**Published:** 2016-12-15

**Authors:** Masoud Rajabioun, Hossein Kazemi Mehrjerdi, Samaneh Ghasemi

**Affiliations:** Department of Clinical Sciences, Faculty of Veterinary Medicine, Ferdowsi University of Mashhad, Mashhad, Iran

**Keywords:** Adactyly, Bilateral, Hemimelia, Hind limbs, Lamb

## Abstract

Hemimelia as a congenital anomaly is a failure of development of extremities formation in embryonic period. This anomaly is defined as complete absence of the part of extremities and different forms were explained for hemimelia. Adactyly is an alternative name for transverse hemimelia and is a rare disorder in the most of animal species. A two months old male lamb with normal vital signs was referred to clinic due to both hind limbs shortness and absence of hooves from the birth day. Clinical and radiological examinations were performed and partial hemimelia was confirmed radiographically in both hind limbs. In left hind limb, total absence of the toe indicated presence of adactyly in this limb. No other congenital deformities were diagnosed in skeletal system based on clinical and radiological examinations. According to our knowledge, this is the first report of such rare conditions in a lamb. Clinical findings and radiological signs of this rare anomaly in a lamb were described in this report.

## Introduction

Congenital anomalies are defects in anatomical structures or functions present at birth.^1^^-^^3^ These abnormalities result from genetic or environmental agents during fetal developmental stages.^1^^,^^2^^,^^4^ Limb abnormality is a form of congenital defect that occurs in numerous manifestations in mammals. The most common sites for these anomalies are distal limbs.^4^^-^^6^ Congenital absence of a portion of extremity is defined as hemimelia that includes several forms. Complete absence of distal portion of the limb is called transverse hemimelia or congenital amputation.^7^ Adactyly is an alternative name represented by total absence of the toe.^3^ It is defined as a congenital anomaly that all or part of digits and their structures such as hooves do not develop.^6^^,^^8^ Although adactyly has been reported in human and animals, but it is a rare condition.^9^

Adactyly as an uncommon anomaly has been reported in human^10^^,^^11^ and different animal species.^9^^,^^12^^-^^19^ In farm animals, it has been reported in shorthorn calf ^12^ and two cases of the Czech cattle population^13^ as well as Southdown lamb.^14^ In carnivores, this anomaly has been shown in cat^15^^,^^16^ and dog.^9^ Adactyly has also been described in Welsh foal,^17^ camel^18^ and donkey.^19^ Information of the only report of adactyly in lamb was not available in detail. Clinical findings and radiological signs of this rare anomaly in a lamb were described in this report.

## Case Description

 A healthy two months old male lamb with congenital anomaly of both hind limbs was referred to the Veterinary Teaching Hospital of Ferdowsi University of Mashhad, Iran. Based on the owner’s information, the lamb was born with abnormal hind limbs. Clinical examinations were performed and the lamb was referred to the radiology department for further evaluations of the affected limbs. Mediolateral and dorsoplantar radiographies (Model Multix Top; Siemens, Dusseldorf, Germany) of the both hind limbs were performed.

**Fig. 1.**
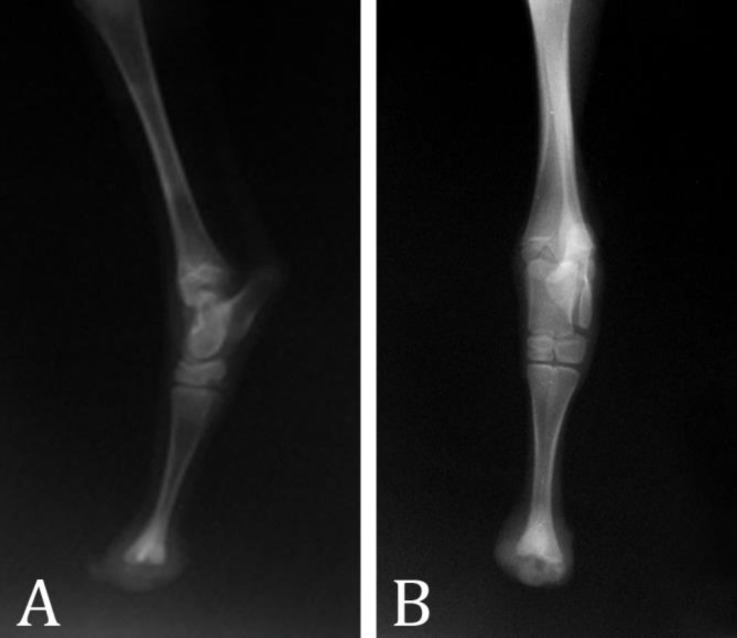
Lateral (A) and dorsoplantar radiograph (B) of the left hind limb, distal of metatarsal bones III and IV were covered by soft tissue opacity and phalanges were not formed

**Fig. 2:**
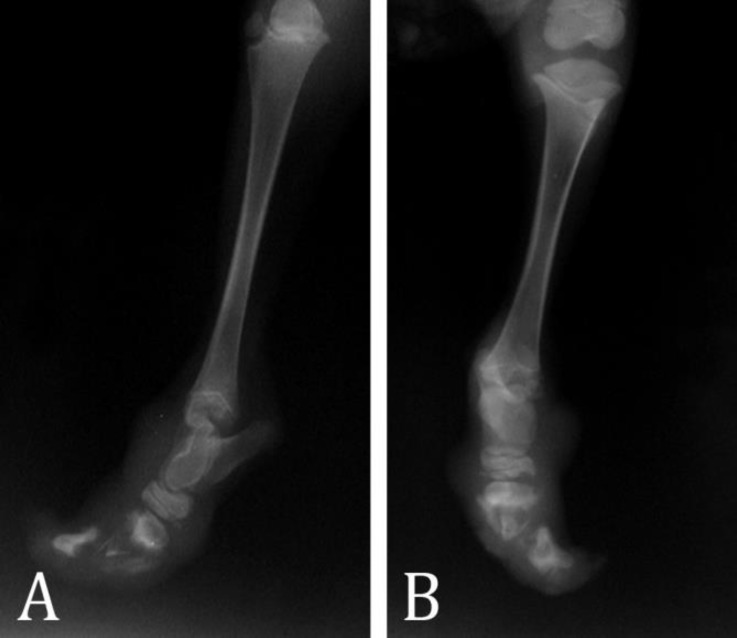
Lateral (A) and dorsoplantar radiograph (B) of the right hind limb, Metatarsal bones III and IV were not formed except the small region in proximal end and separated amorphous bone segments were seen covered by soft tissue opacity

On physical examinations, the lamb was alert and its vital signs were within normal limit. The lamb was standing in oblique manner craniocaudally because of both hind limbs shortness due to absences of distal end of hind limbs. Distal part of the left hind limb was defective at the level of the fetlock joint. In right hind limb, tarsal joint was formed but its distal part was not formed completely. The distal ends of both hind limbs were covered by skin with small amount of keratinized tissue in some regions without skin damage. In spite of the absence of hooves and distal extremities in both hind limbs, the lamb walked normally. No congenital abnormalities were seen in skeletal system in external examinations except of above-mentioned anomalies. Radiography revealed that distal extremities of metatarsal bone III and IV were covered by soft tissue opacity but phalanges were not formed in left hind limb (Fig. 1). In right hind limb, tarsal bone seems to be normal but small abnormal changes in distal row of tarsal bone were not precluded. Metatarsal bone III and IV in right hind limb were not formed except the small region in proximal end. Amorphous separated bone segments were seen covered by soft tissue opacity (Fig. 2). No abnormal radiographic signs were seen in thoracic and abdominal cavities as well as other parts of skeletal system.

## Discussion

 This study described adactyly as an uncommon congenital anomaly in a lamb. In the embryonic stages, hoof originates from modification of epidermal cells situated in skin covering the limbs and finally forms a hard keratinized organ. The hooves have a real structure at the birth and play an important role in protection of the inner tissues.^20^ Congenital limb anomalies occur due to mutation in genes called HOX genes which are important components responsible for interpretation of limbs developmental patterns. Limbs anomalies can be manifested in three different categories including dysplasia, reduction and duplication defects.^8^ Genetic defects, chemotherapeutics (tetracycline, griseofulvin, parabendazole and etc.), drugs such as thalidomide and corticosteroids (in chick embryos), malnutrition (lack of riboflavin), transplacental viral infections and X-ray were considered as causes of congenital anomalies.^21^

Adactyly is marked by absence of fingers and their related structures such as hoof. In ungulates, this defect prevents progress of hoof formation; therefore, presence of skin tissue in distal end of limbs instead of hoof is obvious. Presence of keratin in the hoof structure converts this structure to the suitable surface for bearing the animal weight and protection against surface injuries.^20^ So that, lack of skin replacement with this specialized cornfield tissue can result in many complications such as skin wounds and injuries in underlying tissues. This article reported this defect in a male lamb, although the etiology of adactyly in this case was not obviously clarified.
